# Hydrothermal Synthesized of CoMoO_4_ Microspheres as Excellent Electrode Material for Supercapacitor

**DOI:** 10.1186/s11671-018-2540-3

**Published:** 2018-04-24

**Authors:** Weixia Li, Xianwei Wang, Yanchun Hu, Lingyun Sun, Chang Gao, Cuicui Zhang, Han Liu, Meng Duan

**Affiliations:** Laboratory of Functional Materials and Henan Key Laboratory of Photovoltaic Materials, College of Physics and Materials Science, Henan Normal University, No. 46 Construction East Road, Xinxiang, 453007 Henan China

**Keywords:** Hydrothermal method, CoMoO_4_, Supercapacitor, Electrode material

## Abstract

The single-phase CoMoO_4_ was prepared via a facile hydrothermal method coupled with calcination treatment at 400 °C. The structures, morphologies, and electrochemical properties of samples with different hydrothermal reaction times were investigated. The microsphere structure, which consisted of nanoflakes, was observed in samples. The specific capacitances at 1 A g^−1^ are 151, 182, 243, 384, and 186 F g^−1^ for samples with the hydrothermal times of 1, 4, 8, 12, and 24 h, respectively. In addition, the sample with the hydrothermal time of 12 h shows a good rate capability, and there is 45% retention of initial capacitance when the current density increases from 1 to 8 A g^−1^. The high retain capacitances of samples show the fine long-cycle stability after 1000 charge-discharge cycles at current density of 8 A g^−1^. The results indicate that CoMoO_4_ samples could be a choice of excellent electrode materials for supercapacitor.

## Background

It is important to develop the conversion and storage of renewable alternative energy because of the rapid decay of fossil fuels. Supercapacitor, as a kind of energy storage device, has attracted much attention in recent years [1–5]. Supercapacitors show desirable performance, such as high power density, short charging time and long cycle life [6–8]. According to the mechanism of charge storage, supercapacitors could be classified into electrochemical double-layer capacitors (EDLCs) and redox electrochemical capacitors (i.e. pseudocapacitance (PCs)). The charge storage mechanism of EDLCs is related to the reversible adsorption and desorption of electrolyte ions on electrode/electrolyte surface, whereas that of PCs is related to the redox reactions on the electrode surface [4, 6, 8, 9]. Therefore, whether for EDLCs or PCs, the electrodes are very important, and it is necessary to find an interesting electrode material for supercapacitor application. Generally, the energy density of PCs is higher than that of EDLCs [10, 11]. Many metal oxide materials, such as NiO [12, 13], Co_3_O_4_ [3], CuO [14], MnO_2_ [15], and SnO_2_ [16], have attracted much attention for the use as supercapacitor electrodes. Among these metal oxides, molybdenum oxides and cobalt oxides are the promising candidates for applications due to its high redox activity, multiple oxidation states, high theoretical specific capacitance, reversible small ions storage and low cost [11]. Zhou et al. prepared MoO_2_ nanoparticles, and the sample shows a high specific capacitance of 621 F g^−1^ [17], and Wu et al. investigated the properties of MoO_2_/CNTs with the capacitance of 467.4 F g^−1^ [18].

Mixed metal oxides have attracted much attention because of its high redox activity, good electrical conductivity, reversible small ions storage, and low cost [11]. Among them, the metal molybdates have attracted much attention for energy storage application. Such as NiMoO_4_ [19–21], MnMoO_4_ [22, 23], CoMoO_4_ [6, 8, 11, 24, 25] and other metal molybdates have been extensively investigated as excellent electrode materials for supercapacitor. As reported in Refs. [26–28], CoMoO_4_ is advantageous because of its low cost and non-toxicity and exhibits enhanced electrochemical properties. Veerasubramani et al. prepared the plate-like CoMoO_4_ with a specific capacitance of about 133 F g^−1^ at 1 mA cm^−2^ [26]. Padmanathan et al. synthesized the α-CoMoO_4_ nanoflakes/CFC used as symmetric supercapacitor with a specific capacitance of 8.3 F g^−1^ at a current density of 1 A g^−1^ in organic electrolyte [29]. In addition, Kazemi et al. obtained the dandelion-shape CoMoO_4_ with an excellent specific capacitance of 2100 F g^−1^ at a current density of 1 A g^−1^ [8]. Xia et al. reported that the CoMoO_4_/graphene composites show a specific capacitance of 394.5 F g^−1^ (at the scan rate of 1 mV s^−1^), which is about 5.4 times the value of pure CoMoO_4_ [30].

In this article, the CoMoO_4_ nanoflakes were synthesized by a simple hydrothermal method at different hydrothermal reaction time, followed by calcining at 400 °C in muffle furnace. The electrochemical properties of samples were investigated by using the methods of cyclic voltammetry (CV), galvanostatic charge-discharge (GCD), and electrochemical impedance spectroscopy (EIS). According to GCD test results, the samples show specific capacitances of 151, 182, 243, 384, and 186 F g^−1^ at current density of 1 A g^−1^ in 2 M KOH electrolyte. The sample CMO-12 shows an interesting electrochemical property.

## Experimental

### Synthesis of CoMoO_4_

The CoMoO_4_ samples were synthesized by a simple hydrothermal method. Firstly, 0.4410 g Co(NO_3_)_2_·6H_2_O and 0.2675 g (NH_4_)_6_Mo_7_O_24_·4H_2_O (AHM) were dissolved in 30-mL distilled water with magnetic stirring for 10 min at room temperature to obtain clear mixed solution. Secondly, 0.3621 g urea was slowly added into the mixed solution of Co(NO_3_)_2_·6H_2_O and AHM under magnetic stirring. The mixture was stirred for 1 h to form a homogeneous solution. Next, the homogeneous solution was transferred into a 50-mL Teflon-lined stainless steel autoclave and maintained at 180 °C in an electric oven for 1 h. Other samples were prepared with the hydrothermal times of 4, 8, 12, and 24 h, respectively. The as-synthesized products were cooled to room temperature with the oven. Then, the resulting solution was centrifuged with distilled water and ethanol. The obtained precipitate was dried at 60 °C in vacuum oven for 10 h. Finally, the dried precipitate was calcined at 400 °C in muffle furnace for 2 h to obtain the final products. The final products were marked as CMO-1, CMO-4, CMO-8, CMO-12, and CMO-24, respectively.

### Material Characterization

The crystalline structures of samples were determined by X-ray diffraction (XRD; Bruker, D8 Discover) at 40 kV and 40 mA. The morphologies of samples were examined by field emission scanning electron microscopy (FE-SEM; Zeiss, SUPRA 40) and transmission electron microscopy (TEM; JEM-2100). The nitrogen adsorption-desorption isotherms of samples were obtained by using the Autosorb-iQ physico-adsorption apparatus. Then, the specific surface areas and pore size distributions of samples were obtained by the Brunauer-Emmett-Teller (BET) and Barrett-Joyner-Halenda (BJH) methods.

### Preparation of the Working Electrode and Electrochemical Measurements

The working electrodes were prepared according to the method reported in literature [31]. The as-synthesized products, acetylene blacks, and polytetrafluoroethylene (PTFE) were mixed with a weight ratio of 70:20:10 to form homogeneous paste. Then, it was coated onto the cleaned nickel foam with the area of 1 cm × 1 cm. After drying in a vacuum oven at 50 °C for 6 h to remove the solvent, the nickel foam was then pressed at 10 MPa for 2 min by bead machine. The mass of the active material on the electrode was about 3~5 mg.

The electrochemical properties of samples were characterized by using a CS 350 electrochemical workstation (CorrTest, Wuhan) at room temperature. Two moles per liter of KOH solutions were used as the electrolyte solution, and a three-electrode system was used in the measurement. CoMoO_4_, platinum, and a saturated calomel electrode (SCE) were served as the working electrode, the counter electrode, and the reference electrode, respectively. The CV curves were performed in the potential range of − 0.2 to + 0.6 V at different scan rates of 5, 10, 20, 40, 50, and 100 mV s^−1^. GCD curves were tested at different current densities of 1, 1.5, 2, 3, 5, and 8 A g^−1^. EIS of samples were investigated from 0.01 Hz to 100 kHz.

## Results and Discussion

### Structural and Morphology Characterization

**Fig. 1:**
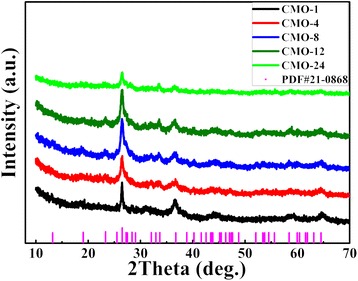
XRD patterns of five samples

As shown in Fig. 1, XRD patterns of samples are consistent with the standard pattern of CoMoO_4_ (JCPDS No. 21-0868), and they are similar as reported in previous [6, 8, 32, 33]. The diffraction peaks at 13.1°, 19.1°, 23.3°, 26.5°, 27.2°, 28.3°, 32.0°, 33.6°, 36.7°, 40.2°, 43.6°, 47.0°, 52.1°, 53.7°, 58.4°, and 64.5° are corresponding to reflections of the (001), ($\overline{2}$01), (021), (002), ($\overline{1}$12), ($\overline{3}$11), ($\overline{1}$31), ($\overline{2}$22), (400), (003), ($\overline{2}$41), (241), ($\overline{2}$04), ($\overline{4}$41), (024), and (243) planes, respectively. As shown in Fig. 1, the broader and weaker diffraction peaks of the XRD patterns for CoMoO_4_ samples were observed, indicating the weaker crystallization in samples. As reported in Refs. [8, 34], the weaker crystallinity plays a critical role for enhancing the electrochemical behavior in supercapacitor applications.

**Fig. 2:**
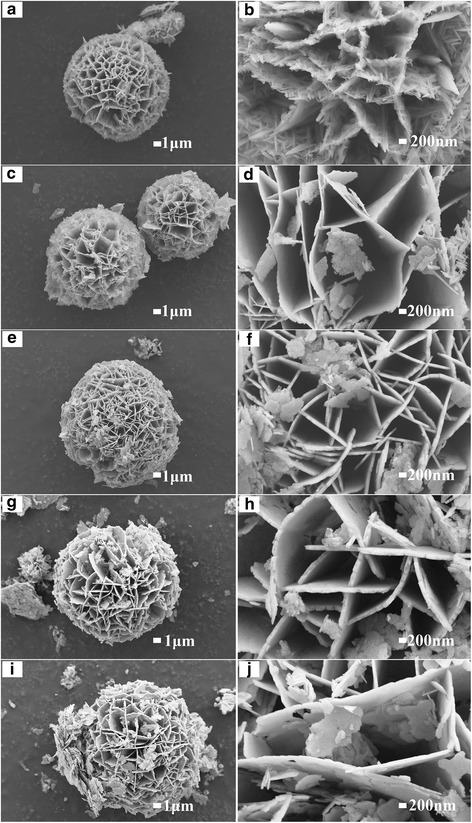
The low and high-magnification SEM images of samples. **a**, **b** CMO-1. **c**, **d** CMO-4. **e**, **f** CMO-8. **g**, **h** CMO-12. **i**, **j** CMO-24

**Fig. 3:**
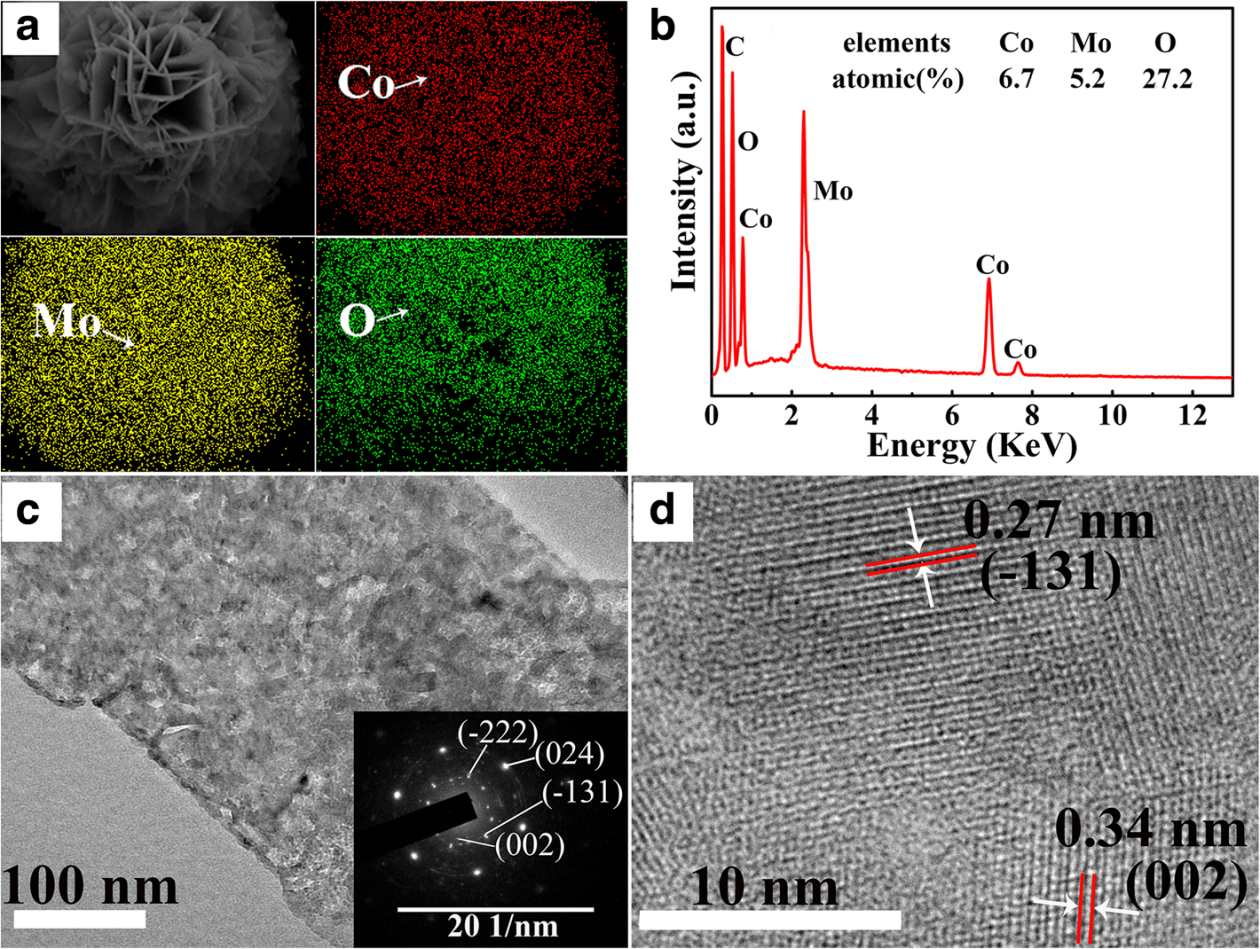
**a** Energy-dispersive spectroscopy (EDS) element mapping image and Co, Mo, and O distribution. **b** The EDS spectrum and inset are molar ration percent of Co, Mo, and O elements. **c** High-magnification TEM and inset are the selected area electron diffraction pattern. **d** The HRTEM image of CMO-12 sample

The surface morphologies of CoMoO_4_ samples were characterized by SEM and TEM. As shown in Fig. 2, microsphere structures were observed for all samples, and the microsphere consisted of nanoflakes. With the increase of hydrothermal time, the thickness of nanosheetsincreases first and then decreases, and the thickest nanoflakes were obtained in sample with the hydrothermal time of 12 h. Figure 3a, b show the energy-dispersive spectroscopy (EDS) element mapping images and EDS spectrum of CMO-12. According to the element mapping images, Co, Mo, and O elements uniformly distributed in the microsphere. The element molar ratio of Co, Mo, and O is about 1:1:4, which is corresponding to the composition of CoMoO_4_. Figure 3c, d show the TEM images of the CMO-12. As shown in the inset of Fig. 3c, the selected area electron diffraction (SAED) patterns reveal the single-crystalline nature of the CoMoO_4_. The clear diffraction spots could be assigned to the ($\overline{2}$22), (024), ($\overline{1}$31), and (002) crystal planes of the CoMoO_4_. Figure 3d is the HRTEM image; it shows the lattice spacing of 0.34 and 0.27 nm, which could be related to the (002) and ($\overline{1}$31) planes of CoMoO_4_, respectively.

**Fig. 4:**
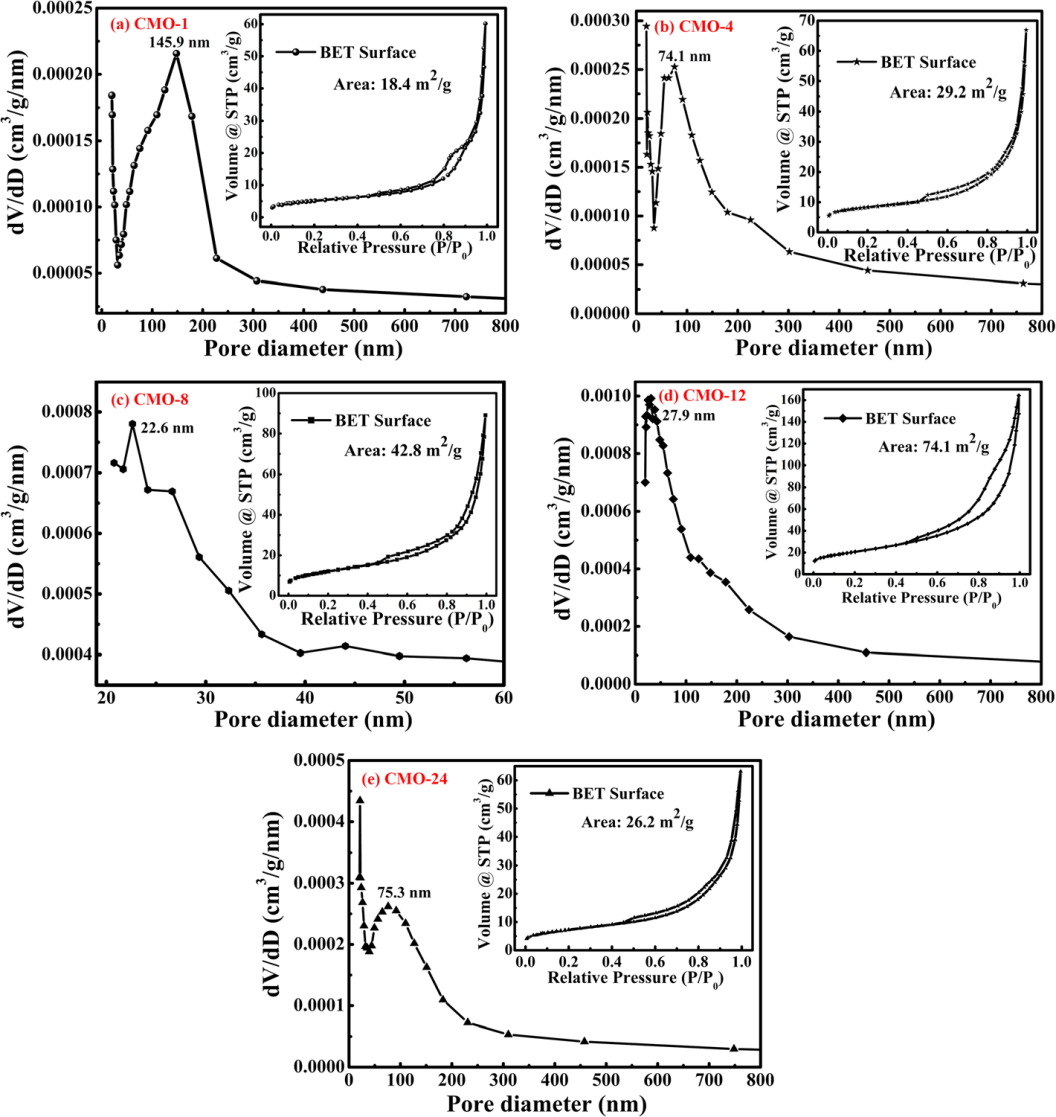
Pore size distributions and N_2_ adsorption-desorption isotherms of samples

Figure 4 shows the pore size distributions and N_2_ adsorption-desorption isotherms of CoMoO_4_ samples. According to the N_2_ adsorption-desorption isotherms of samples, all the isotherms show the feature of type IV with H_3_-type hysteresis loops. The BET specific surface areas of CMO-1, CMO-4, CMO-8, CMO-12, and CMO-24 were calculated to be 18.4, 29.2, 42.8, 74.1, and 26.2 m^2^ g^−1^, respectively. Sample CMO-12 shows the highest BET surface area, and the high BET surface area could increase the contact area of electrode/electrolyte and provide more active sites for efficient transport of electrons and ions in electrode system [35]. As shown in Fig. 4, sharp peaks in pore size distributions of samples are located at 145.9, 74.1, 22.6, 27.9, and 75.3 nm, respectively. It indicates that there are mesopores in CMO-8 and CMO-24. However, a few macropores are detected in samples CMO-1, CMO-4, and CMO-24. When materials are used in supercapacitors, mesopore structures of materials also could increase the contact area between electrode and electrolyte; there are more sufficient active sites for efficient transport of electrons and ions in electrode system [36–38]. Therefore, CMO-12 with highest BET surface area and mesopore structure might show better electrochemical properties than other samples.

### Electrochemical Characterization

**Fig. 5:**
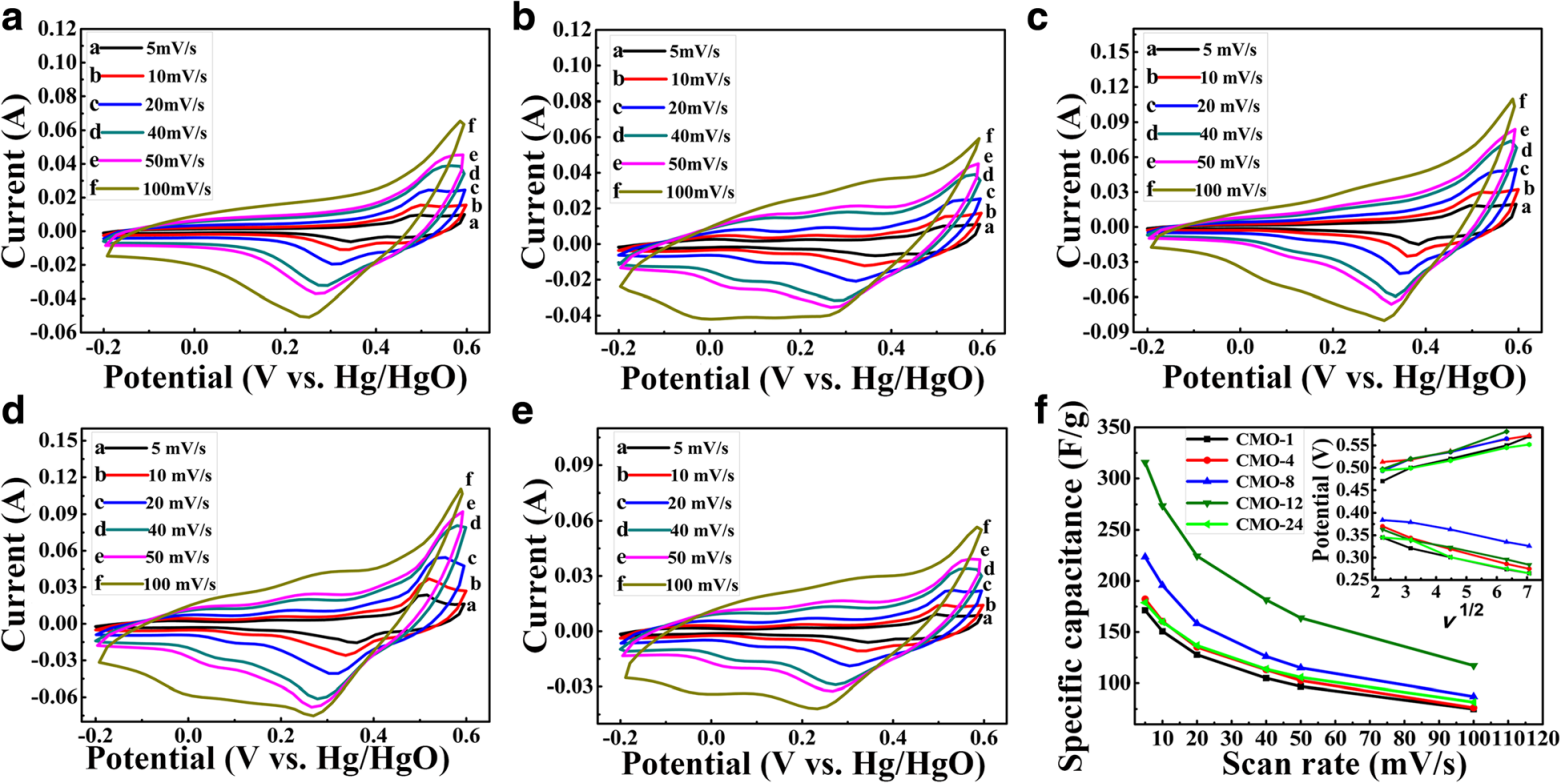
The CV curves under different scan rates of 5, 10, 20, 40, 50, and 100 mV s^−1^ of samples **a** CMO-1, **b** CMO-4, **c** CMO-8, **d** CMO-12, and **e** CMO-24 in the potential window ranged from − 0.2 to + 0.6 V. **f** The specific capacitance of samples under different scan rates of 5–100 mV s^−1^. The inset in **f** is the plots of anodic and cathodic peak current against the square root of the scan rate

CV curves of CoMoO_4_ samples at different scan rates of 5–100 mV s^−1^ in 2 M KOH electrolyte with potential range from − 0.2 V to + 0.6 V (vs. Hg/HgO) are shown in Fig. 5a –e. The typical Faradic reaction peaks can be clearly seen in all curves, which indicate that the CoMoO_4_ electrodes are pseudocapacitor electrodes. The observed redox peak is due to the charge-transfer kinetics of Co^2+^ and Co^3+^ associated with the OH^−^ in electrolyte [8, 26]. The redox reaction of Co^2+^/Co^3+^ is listed as follows [39, 40]:

1 $$ {\mathrm{CoMoO}}_4+{\mathrm{OH}}^{-}\to \mathrm{CoOOH}+{\mathrm{MoO}}_3+{\mathrm{e}}^{-} $$

2 $$ \mathrm{CoOOH}+{\mathrm{OH}}^{-}\kern0.28em \iff \kern0.28em {\mathrm{CoO}}_2+{\mathrm{H}}_2\mathrm{O}+{\mathrm{e}}^{-} $$

3 $$ \mathrm{CoOOH}+{\mathrm{H}}_2\mathrm{O}+{\mathrm{e}}^{-}\kern0.28em \iff \kern0.28em \mathrm{Co}{\left(\mathrm{OH}\right)}_2+{\mathrm{OH}}^{-} $$

As shown in Fig. 5a–e, with the increase of scan rates, the redox peaks shift to higher and lower potentials, respectively. The potential difference between oxidation peaks and reduction peaks also increased with the increased scan rate. It indicates that the irreversible degree and the quasi-reversible reaction are increased with the increase of scan rate [36, 41]. The shift is mainly related to the internal resistance of the electrode and the polarization caused by high scan rate [36, 42]. A near linearly relationship between redox peaks potentials and the square root of the scan rate was observed, which can be seen in the inset of Fig. 5f. The approximately linear relationship also indicates that the reaction kinetics during redox process is probably controlled by ions diffusion process [6].

According to the CV curves, the specific capacitance of samples can be calculated by the following equation:

4 $$ {C}_{\mathrm{sp}}=\frac{\int_{V_1}^{V_2} IdV}{m\times v\times \Delta V}, $$

where *C*_sp_ (F g^−1^) is the specific capacitance, *V*_1_ and *V*_2_ are the start and end voltage, ∫*IdV* is the integral area of CV curves, *m* (g) is the mass of active materials loading on the electrode, *v* (mV s^−1^) is the potential scan rates, and ∆*V* (V) is the sweep potential window. The specific capacitances of samples were calculated based on the CV curves by using Eq. (4), which are shown in Fig. 5f. The specific capacitances of all samples decrease as the increase of scan rates. More OH^−^can reach the active site with more favorable conditions at low potential scan rate [31, 43]. Furthermore, a higher scan rate leads to either depletion or saturation of the protons in the electrolyte inside the electrode during the redox process, and only the outer surface could be utilized for the charge storage [41, 43, 44]. When the hydrothermal synthesis time increases from 1 to 12 h, the specific capacitances of samples show an obviously increase from 171.3 to 315.7 F g^−1^ at a scan rate of 5 mV s^−1^. However, the specific capacitance decreases from 315.7 to 178.7 F g^−1^ when the hydrothermal time increases from 12 to 24 h. Therefore, the CMO-12 sample (i.e., the hydrothermal time is 12 h) shows an excellent specific capacitance. The specific capacitance of 315.7 F g^−1^ at 5 mV s^−1^ is better than that of 286 F g^−1^ for CoMoO_4_ nanorods [11] and 95.0 F g^−1^ for pure CoMoO_4_ [45] and comparable with 322.5 F g^− 1^ for RGO/CoMoO_4_ [45].

**Fig. 6:**
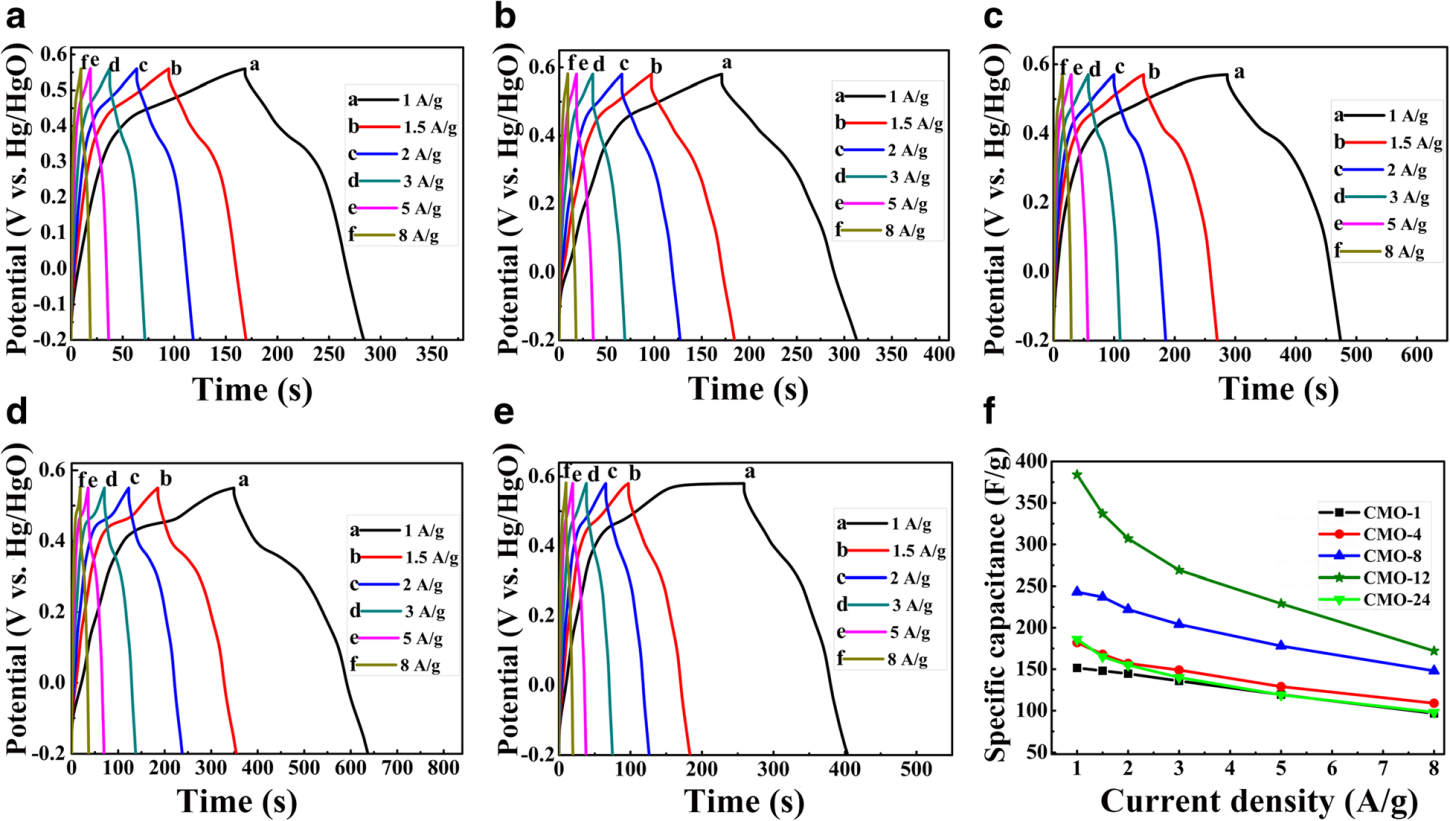
The GCD curves under different current densities of 1, 1.5, 2, 3, 5, and 8 A g^−1^ of samples **a** CMO-1, **b** CMO-4, **c** CMO-8, **d** CMO-12, and **e** CMO-24 in the potential window ranged from − 0.2 to + 0.58 V. **f** The specific capacitance of samples calculated by GCD results

Such an improved electrochemical property can be confirmed by the following galvanostatic charge-discharge tests. The GCD tests of samples were performed at different current densities of 1, 1.5, 2, 3, 5, and 8 A g^−1^ in 2 M KOH electrolyte, and the results are shown in Fig. 6a–e. The nonlinear GCD curves could be attributed by the redox reaction [46], and this is consistent with the CV curves. As shown in these curves, the discharge time of CMO-12 is significantly longer than other samples, indicating a much higher specific capacitance in CMO-12. This could be confirmed furtherly by the following calculated results. The specific capacitances of CoMoO_4_ electrode can be calculated by using the equation as follow:

5 $$ C=\frac{I\times \Delta t}{m\times \Delta V}, $$

where *C* (F g^−1^) is the specific capacitance, *I* (A) is the discharge current, ∆*t* (s) is refer to discharge times, *m* (g) is the mass of active material loading on the electrode surface, and ∆*V* (V) is the applied potential window [6, 8, 26]. Figure 6f shows the calculated specific capacitance of samples at different current densities. With the increase of current density, the specific capacitances of samples are decreased. This can be attributed to the effective contact between ions and electro-active sites of electrode. At high current density, there is only part of the total available reaction sites because the electrolyte ions suffer from low diffusion, which lead to an incomplete insertion reaction and a low specific capacitance [19, 45]. From Fig. 6f, we can see that the CMO-12 has the highest specific capacitance, which are 384, 337, 307, 269, 229, and 172 F g^−1^ at the current density of 1, 1.5, 2, 3, 5, and 8 A g^−1^, respectively. The specific capacitance of CMO-12 shows a good rate capability. Furthermore, the specific capacitance of CMO-12 is also higher than that reported in some previous literatures. As reported by Tian et al. [39], the specific capacitance of needle-like Co-Mo-O is 302 F g^−1^ at a current density of 1 A g^−1^. The maximum specific capacitance of CoMoO_4_ was about 133 F g^−1^ at 1 mA cm^−2^ in the article of Veerasubramani [26]. In Ref. [29], the specific capacitance of α-CoMoO_4_ nanoflakes/CFC used as symmetric supercapacitor is only 8.3 F g^−1^ at current density of 1 A g^−1^. Besides, a high discharge rate or a high current density is very important for a real supercapacitor device, which involves a fast charging-discharging process [43]. At a high current density of 8 A g^−1^, the specific capacitances for the five samples are 97, 109, 148, 172, and 98 F g^−1^, respectively.

**Fig. 7:**
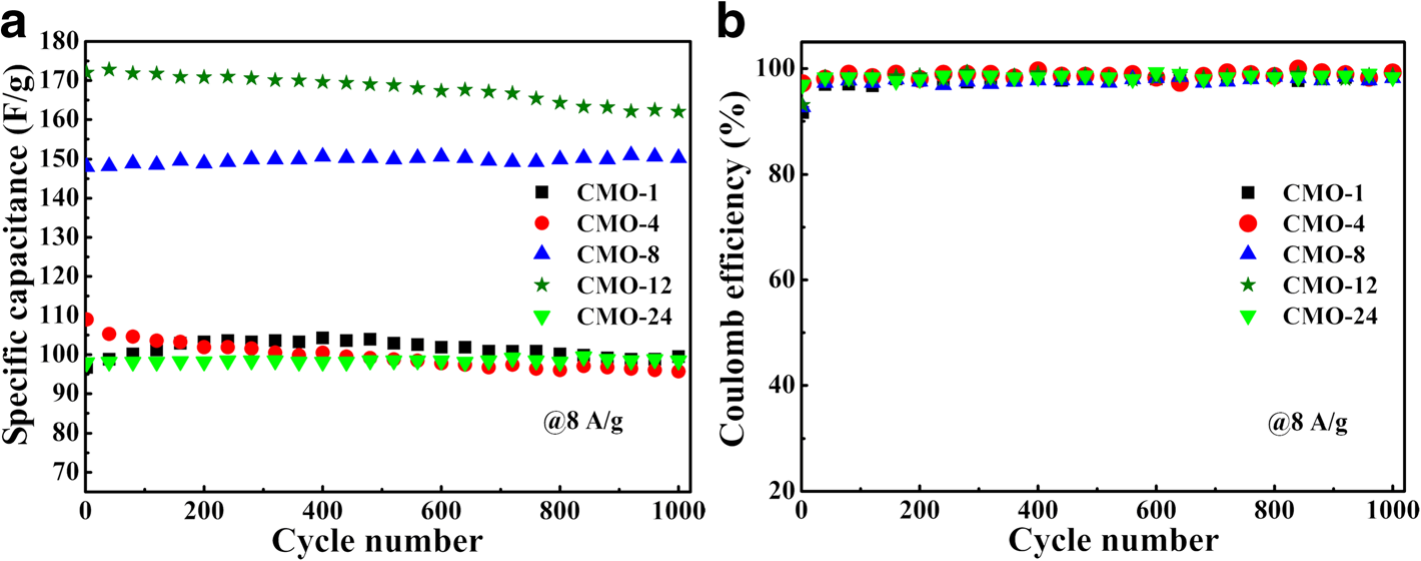
**a** Cycling performance of the electrodes at 8 A g^−1^. **b** The corresponding coulombic efficiency of samples during cycling test

The stabilities of the CoMoO_4_ electrodes were detected in 2 M KOH electrolyte at a current density of 8 A g^−1^ for 1000 cycles, which are shown in Fig. 7. After 1000 cycles, the five samples show the retention of 102.9, 87.8, 101.5, 94.2, and 100.5%, respectively. For the increase of specific capacitance during the cyclic charge-discharge process, it could be ascribed to activation of the CoMoO_4_ surface with time [6]. It makes the surface of CoMoO_4_ contact fully with the electrolyte, which leads to the improvement of electrochemical property [6, 47, 48]. Figure 7b shows the coulombic efficiency of CoMoO_4_ samples during the 1000 charge-discharge cycles, which also shows high specific capacitance. The results indicate that all these samples have fine long-cycle stability. The highly specific capacitance, great rate capacity, and fine long-cycle stability indicate that the CMO-12 sample has an excellent electrochemical property.

**Fig. 8:**
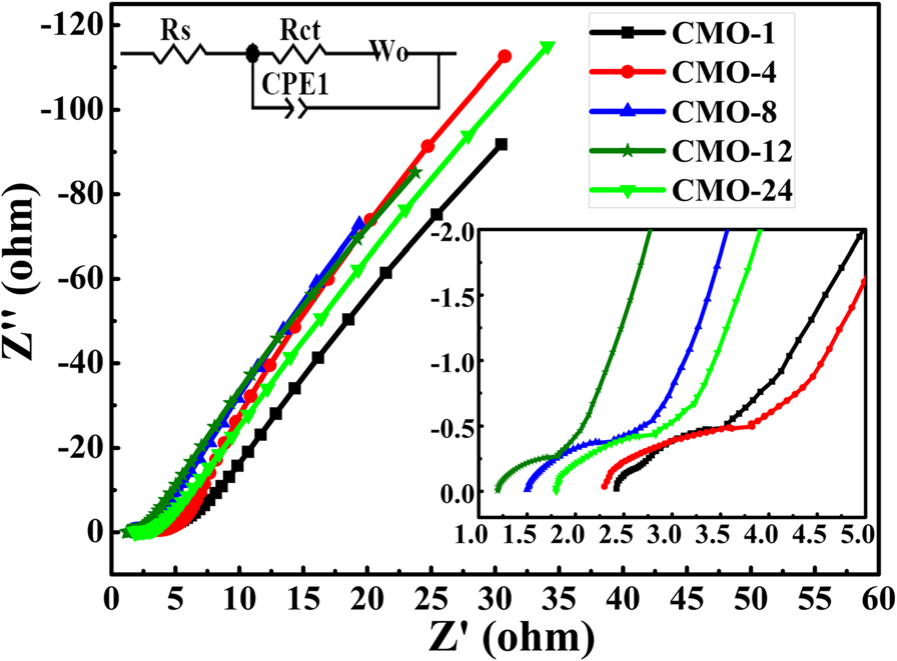
EIS spectrums of samples obtained at the frequency ranging from 0.01 Hz to 100 KHz. The inset is the local amplification of EIS spectrums and the equivalent circuit diagram

To further evaluate the electrochemical property of CoMoO_4_ electrode, the EIS of five samples are recorded in 2 M KOH electrolyte. Figure 8 is the Nyquist plots of five samples. The Nyquist plots represent the frequency response of the electrode/electrolyte system [26, 49]. The EIS spectra can be fitted by the equivalent circuit diagram, which was inserted in Fig. 8. The Nyquist plot is composed of a semicircle at high frequency and a straight line at low frequency. The semicircle diameter at high frequency represents the Faraday interface charge transfer resistance (*R*_ct_), and the slope of the straight line at low frequency is the representative of the typical Warburg resistance (W_0_) [41], respectively. CPE1 is a constant phase element, accounting for the double-layer capacitance [43]. In addition, the series resistance *R*_s_ is the internal resistance, which could be obtained from the intercept of the plots on the real axis [11]. The measured *R*_s_ values are 2.83, 2.41, 1.51, 1.22, and 2.26 Ω for the five samples, respectively. And the fitted *R*_ct_ values of the five samples are1.69, 1.48, 0.72, 0.23, and 1.28 Ω. The EIS results show that the CMO-12 sample has lower values of *R*_s_ and *R*_ct_ than the other four samples. This indicates that the CMO-12 sample has higher electronic and ionic conductivities than the other samples [35, 50, 51]. Besides, CMO-12 with mesopores structure has higher BET surface area than the other samples. The high BET surface area and good conductivity are benefic for redox reaction in electrode/electrolyte system.

## Conclusions

In summary, the CoMoO_4_ microspheres have been successfully synthesized by hydrothermal growth process coupled with calcinations treatment. The hydrothermal synthesis times are 1, 4, 8, 12, and 24 h, respectively. XRD patterns indicate that single phase CoMoO_4_ structure was obtained. SEM images show the microspheres were composed of nanoflakes. The CMO-12, which was prepared with the hydrothermal time of 12 h, has demonstrated an excellent supercapacitor performance. According to GCD tests, the specific capacitances of CMO-12 are 384, 337, 307, 269, 229, and 172 F g^−1^ at current densities of 1, 1.5, 2, 3, 5, and 8 A g^−1^, respectively, while it just reached 151, 182, 243, or 186 F g^−1^ at the current density 1 A g^−1^ for other samples with different hydrothermal times. The retain capacitances of CMO-12 sample after 1000 charging-discharging cycles at current density of 8 A g^−1^ show the fine long-cycle stability. Such excellent capacitive behavior could be ascribed to the microsphere structure and high BET surface area, and the good conductivity in CMO-12 electrode is also helpful to the improvement of capacitive behavior. The high specific capacitance, good rate capability, and excellent cycling stability promote the practical application of CoMoO_4_ materials in supercapacitors.

## Abbreviations

BET: Brunauer-Emmett-Teller
CV: Cyclic voltammetry
EDS: Energy-dispersive spectroscopy
EIS: Electrochemical impedance spectroscopy
FE-SEM: Field emission scanning electron microscopy
GCD: Galvanostatic charge-discharge
PTFE: Polytetrafluoroethylene
SAED: Selected area electron diffraction
SCE: Saturated calomel electrode
TEM: Transmission electron microscopy
XRD: X-ray diffraction
